# Advances in Gouty Arthritis Management: Integration of Established Therapies, Emerging Treatments, and Lifestyle Interventions

**DOI:** 10.3390/ijms251910853

**Published:** 2024-10-09

**Authors:** Ting-Kuo Yao, Ru-Ping Lee, Wen-Tien Wu, Ing-Ho Chen, Tzai-Chiu Yu, Kuang-Ting Yeh

**Affiliations:** 1 Department of Orthopedics, Hualien Tzu Chi Hospital, Buddhist Tzu Chi Medical Foundation, Hualien 970473, Taiwan; tayao0318@tzuchi.com.tw (T.-K.Y.); timwu@tzuchi.com.tw (W.-T.W.); ihchen@tzuchi.com.tw (I.-H.C.); feyu@tzuchi.com.tw (T.-C.Y.); 2 Institute of Medical Sciences, Tzu Chi University, Hualien 970374, Taiwan; fish@gms.tcu.edu.tw; 3 School of Medicine, Tzu Chi University, Hualien 970374, Taiwan; 4 Graduate Institute of Clinical Pharmacy, Tzu Chi University, Hualien 970374, Taiwan

**Keywords:** gouty arthritis, urate-lowering therapy, anti-inflammatory agents, phytotherapy, complementary therapies, canakinumab, *Citrullus colocynthis*, oxidative stress, lifestyle modifications, personalized medicine

## Abstract

Gouty arthritis, a prevalent inflammatory condition characterized by the deposition of monosodium urate crystals within joints, often results in debilitating pain and inflammation. Conventional therapeutic approaches, including nonsteroidal anti-inflammatory drugs, corticosteroids, and urate-lowering agents such as allopurinol and febuxostat, often have limitations such as adverse effects, drug interactions, and suboptimal patient compliance. This review presents a comprehensive overview of both established and emerging therapeutic strategies, developed between 2019 and 2024, for gouty arthritis; the review focuses on their mechanisms of action, efficacy, and safety profiles. Novel therapeutic approaches include pharmaceutical plant additives (e.g., *Citrullus colocynthis*, *Atractylodes lancea*), anti-inflammatory agents such as canakinumab and ozone therapy, and complementary therapies such as warm ginger compresses, Qingpeng ointment, and various lifestyle modifications. These strategies offer promising alternatives to conventional treatments by targeting uric acid metabolism, inflammatory pathways, and crystal formation, potentially reducing reliance on standard medications and minimizing adverse effects. Although therapies such as canakinumab have demonstrated significant efficacy in reducing gout flares, others such as polyphenol-rich foods offer favorable safety profiles. Further research, including large-scale clinical trials, is warranted to validate these findings and integrate these strategies into clinical practice to improve patient outcomes and quality of life.

## 1. Introduction

Gouty arthritis is a common form of inflammatory arthritis characterized by acute and severe pain, redness, and swelling in the joints. It has become a major public health concern owing to its increasing prevalence, particularly in developed countries, and its association with various comorbidities such as cardiovascular disease, chronic kidney disease, and metabolic syndrome [1]. The condition primarily results from hyperuricemia, an elevated concentration of uric acid in the blood, which precipitates the formation and deposition of monosodium urate (MSU) crystals in the joints and adjacent tissues, triggering intense inflammatory responses [2].

Biochemistry of Uric Acid Production: Uric acid is the final oxidation product of purine metabolism in humans. Unlike most mammals, humans lack the enzyme uricase, which catalyzes the degradation of uric acid into the more soluble allantoin. This evolutionary loss of uricase predisposes humans to hyperuricemia due to the relatively high concentration of uric acid in the bloodstream [3]. The synthesis of uric acid begins with the degradation of purine nucleotides. Dietary purines and those derived from the normal turnover of nucleic acids are converted into hypoxanthine, which is subsequently oxidized by xanthine oxidase to xanthine and ultimately to uric acid. The overproduction or underexcretion of uric acid leads to hyperuricemia, the primary risk factor for gout [4].

Effects and Consequences of Hyperuricemia: Hyperuricemia can result from overproduction of uric acid, impaired renal excretion, or a combination of these factors. Renal excretion accounts for approximately two-thirds of daily uric acid elimination, and the gastrointestinal tract accounts for the remaining one-third [5]. MSU crystals are deposited within joints and tissues when the serum urate concentration exceeds its solubility threshold, which is approximately 6.8 mg/dL at physiological pH and temperature. These crystals can activate the NLRP3 inflammasome within macrophages, leading to the cleavage and release of the proinflammatory cytokine interleukin-1 beta (IL-1β). IL-1β further promotes the release of other cytokines, such as tumor necrosis factor-alpha (TNF-α) and interleukin-6 (IL-6), and the recruitment of neutrophils, perpetuating the inflammatory response and causing the severe pain and swelling characteristic of gout flares [6].

Pathophysiology of Gouty Arthritis: Gouty arthritis is characterized by a complex interplay between metabolic abnormalities and inflammatory processes. When MSU crystals are deposited in the synovial fluid, they are phagocytosed by resident macrophages and synoviocytes, triggering the activation of the NLRP3 inflammasome. This activation promotes the caspase-1-dependent maturation and secretion of IL-1β, a critical mediator of the acute inflammatory response in gout [7]. Furthermore, the interaction between MSU crystals and Toll-like receptors (TLR2 and TLR4) on immune cells stimulates the release of additional inflammatory mediators, including chemokines that attract neutrophils to the inflammation site [8]. Neutrophils in turn contribute to the formation of a proinflammatory milieu by releasing reactive oxygen species, proteolytic enzymes, and other cytokines, further amplifying tissue damage and prolonging inflammation [9]. Chronic inflammation and recurrent gout attacks can result in tophaceous gout, a more severe form of the disease characterized by the deposition of MSU crystals in soft tissues and the formation of tophi. Tophi can lead to joint destruction, deformities, and functional impairment.

Molecular Pathways and Genetic Factors: Recent research has elucidated several genetic factors and molecular pathways involved in the development and progression of gout. Genetic polymorphisms in genes encoding urate transporters, such as SLC2A9 (GLUT9) and ABCG2, have been associated with altered renal urate excretion and an increased risk of hyperuricemia and gout [10]. Additionally, the role of the renin–angiotensin system, oxidative stress, and endothelial dysfunction in the pathogenesis of hyperuricemia has garnered research attention. Hyperuricemia-induced oxidative stress can impair endothelial function by reducing nitric oxide (NO) bioavailability, contributing to the development of hypertension and cardiovascular disease, which are frequently comorbid with gout [11]. Understanding these molecular and genetic mechanisms provides valuable insights into potential therapeutic targets and personalized treatment approaches for gout management.

Rationale for Exploring New Therapeutic Approaches: Conventional therapeutic approaches for treating gouty arthritis, including nonsteroidal anti-inflammatory drugs (NSAIDs), corticosteroids, colchicine, and urate-lowering therapies (ULTs) such as allopurinol and febuxostat, primarily target symptom management rather than the biochemical pathways underlying hyperuricemia and inflammation [12]. These therapies often have limitations, including adverse side effects, drug–drug interactions, and poor patient compliance. Moreover, many patients, particularly those with comorbid conditions, fail to achieve adequate serum urate control or continue to experience frequent gout flares despite conventional treatment [7].

Considering these challenges, the identification of innovative therapeutic strategies that directly target the specific biochemical and molecular pathways involved in uric acid metabolism, inflammatory mediator production, and MSU crystal formation is imperative. With the rapid advances in our understanding of the complex mechanisms underlying gout, researchers are developing novel therapies that offer more targeted and effective management options, potentially improving patient outcomes and reducing the disease burden.

For this narrative review, we conducted a comprehensive search of the following databases: PubMed, MEDLINE, Embase, and Cochrane Library. These databases were selected to ensure a broad range of high-quality studies, including clinical trials, systematic reviews, meta-analyses, and relevant articles published between 2019 and 2024. Specific search terms related to “gouty arthritis”, “urate-lowering therapies”, “anti-inflammatory treatments”, “phytotherapy”, “complementary therapies”, and “lifestyle modifications” were used to identify the relevant literature. A comprehensive summary of both established and emerging treatment methods for gouty arthritis has been summarized in Table 1.

The aim of the review was to provide a comprehensive overview of both traditional and emerging therapeutic approaches to gouty arthritis, including well-established medical treatments, alternative therapies, and lifestyle modifications.

## 2. Uric Acid-Lowering Therapies

We explored various therapeutic strategies, including dietary modifications, medicinal treatments, and pharmaceutical plant additives, for effectively lowering serum uric acid levels, thereby preventing MSU crystal formation. A study revealed the importance of maintaining serum urate levels below 6.8 mg/dL to avoid urate crystal precipitation [48].

### 2.1. Dietary Strategies

#### 2.1.1. Reducing Purine-Rich Foods

A diet low in purine can result in a significant reduction in serum urate levels. Purine-rich foods, such as red meat, organ meats (e.g., liver and kidney), shellfish, and certain types of seafood (e.g., sardines, anchovies, and mackerel), are metabolized into uric acid. Reducing the consumption of these foods helps lower uric acid production in the body, thereby reducing the risk of gout flares. Accordingly, patients are advised to consume lean meats, poultry, and plant-based protein sources such as legumes, which have lower purine content [49].

#### 2.1.2. Limiting Alcohol Consumption

Alcohol, particularly beer and spirits, has been strongly associated with increased uric acid levels because of its high purine content and its ability to reduce renal uric acid excretion. Beer contains guanosine, a nucleotide that is metabolized into uric acid, and spirits can increase the production of lactic acid, which competes with uric acid for renal excretion. Limiting alcohol intake, particularly beer, and consuming wine (which has a lower purine content) may moderately help reduce serum urate levels [50].

#### 2.1.3. Increasing Consumption of Low-Purine and Low-Fat Dairy Products

Low-fat dairy products such as skim milk and yogurt have been demonstrated to reduce serum urate levels and the risk of gout flares. The uricosuric effect of dairy is attributable to its ability to promote uric acid excretion and its high content of orotic acid, which reduces the reabsorption of uric acid in the renal tubules. The literature demonstrates that regular consumption of low-fat dairy can reduce the risk of gout and recurrent flares [51].

#### 2.1.4. Incorporating Polyphenol-Rich Foods

Recent research has demonstrated that food polyphenols have the potential to manage gouty arthritis because of their anti-inflammatory, antioxidant, and uric acid-lowering properties [52]. These natural compounds exist in various fruits, vegetables, and plant-based foods and have been identified as promising alternatives or adjunct treatments. Polyphenols such as flavonoids, phenolic acids, stilbenes, and lignans can inhibit proinflammatory pathways and cytokines involved in gouty inflammation, such as TNF-α, IL-1β, and IL-6 [53]. For example, quercetin, a well-known flavonoid, effectively reduces the levels of these inflammatory mediators during gout attacks. Zou et al. reviewed the mechanisms through which food polyphenols modulate inflammatory processes and reduce oxidative stress, and their results revealed that these compounds inhibit nuclear factor-κB activation, a critical transcription factor involved in cytokine production, thereby reducing gout-associated inflammation and pain [54].

Polyphenols, such as resveratrol and epigallocatechin gallate, possess potent antioxidant capacities that have been reported to neutralize free radicals, reduce oxidative stress, and enhance endogenous antioxidant defenses, which may be particularly beneficial in gout management [55]. However, the evidence supporting their efficacy remains mixed. Resveratrol, a polyphenolic compound found in grapes and red wine, has been explored for its potential anti-inflammatory and cardioprotective effects. Recent studies suggest that resveratrol does not significantly reduce inflammatory substances in the blood nor does it have a substantial positive impact on cardiovascular risks, casting uncertainty on its role in managing inflammation and cardiovascular complications in gout. Thus, more large-scale, well-designed clinical trials are needed to clarify the therapeutic potential of resveratrol in this context. Other polyphenol-rich compounds, such as those found in cherries and berries, have shown promise in gout management by inhibiting xanthine oxidase, thereby reducing uric acid synthesis. Clinical studies have demonstrated that regular consumption of tart cherry juice significantly lowers serum uric acid levels and reduces the frequency of gout flares [56]. These findings suggest that incorporating polyphenol-rich foods into the diet could be beneficial for managing hyperuricemia and preventing gout attacks.

However, while these results are promising, much of the current evidence on polyphenols comes from small-scale trials or observational studies that often lack rigorous methodological designs, such as randomized controlled trials (RCTs). Although the ease of integrating food polyphenols into the diet may enhance patient compliance compared with conventional medications, the potential interactions with other medications, as well as the optimal dosages and combinations, remain unclear. Moreover, while food polyphenols are generally considered safe and may offer broader health benefits, such as cardiovascular protection and anticancer properties [57], variability in study quality and the lack of standardized dosing regimens highlight the need for further investigation. To establish clear evidence-based guidelines, large-scale, well-designed RCTs are warranted to confirm the efficacy, safety, and therapeutic potential of specific polyphenols in gout management.

#### 2.1.5. Enhancing Hydration

Adequate hydration is essential in promoting renal clearance of uric acid. Increased water intake dilutes the concentration of uric acid in the urine, reducing the risk of crystal formation. Patients with gout are advised to consume at least 2–3 L of water per day, particularly during acute flares or when engaging in activities that cause sweating. A study reported that individuals who consumed at least eight glasses of water daily exhibited a lower risk of gout attacks than did those who consumed fewer glasses [58].

#### 2.1.6. Consuming Foods Rich in Vitamin C

Vitamin C has been demonstrated to reduce serum urate levels by enhancing uric acid excretion through its uricosuric effects. Vitamin C competes with uric acid for reabsorption in renal tubules, leading to increased urinary excretion. Consuming 500 mg of vitamin C daily has been associated with a modest but significant reduction in serum urate levels. The incorporation of foods rich in vitamin C, such as oranges, strawberries, bell peppers, and broccoli, into the diet is recommended to support gout management [59].

#### 2.1.7. Limiting Fructose and Sugar-Sweetened Beverages

Fructose is a simple sugar found in many processed foods and sugary beverages; it is metabolized into uric acid in the liver. High fructose intake increases uric acid production and is strongly associated with a relatively high risk of gout. Reducing the consumption of sugar-sweetened beverages, such as soft drinks and fruit juices, and avoiding high-fructose corn syrup can help reduce uric acid levels. Patients are encouraged to choose whole fruits over fruit juices to minimize fructose intake [60].

### 2.2. Medicinal Treatments (Topical and Systemic)

#### 2.2.1. Allopurinol (Systemic)

Allopurinol is the most commonly employed first-line therapy for gout; it can effectively reduce serum urate levels in most patients by inhibiting xanthine oxidase. However, its dose must be meticulously adjusted on the basis of renal function to minimize the risk of severe hypersensitivity reactions, including allopurinol hypersensitivity syndrome (AHS), which can lead to rashes, hepatitis, interstitial nephritis, and potentially fatal outcomes [13]. Genetic screening is recommended for high-risk populations, particularly those with the HLA-B*5801 allele, such as patients of Asian descent, to mitigate the risk of AHS [14]. Although allopurinol is effective, careful monitoring of patients with renal impairment is crucial to avoid adverse events.

#### 2.2.2. Febuxostat (Systemic)

Febuxostat, a newer xanthine oxidase inhibitor, is an alternative for patients who are intolerant to or unresponsive to allopurinol. In contrast to allopurinol, febuxostat does not require dose adjustments for mild-to-moderate renal impairment, rendering it a more suitable option for patients with reduced kidney function [15]. Clinical trials have demonstrated that the effectiveness of febuxostat is comparable to that of allopurinol in reducing serum urate levels. However, the CARES trial reported that febuxostat use was associated with an increased risk of cardiovascular events [16]. Therefore, febuxostat is generally reserved for patients who are intolerant to allopurinol or for whom allopurinol is contraindicated; careful monitoring is recommended for those with a history of cardiovascular disease.

#### 2.2.3. Probenecid (Systemic)

Probenecid, a well-established uricosuric agent, is particularly suitable for patients who cannot tolerate or do not respond adequately to xanthine oxidase inhibitors. Probenecid can be used alone or in combination with allopurinol or febuxostat to achieve target serum urate levels. However, probenecid requires adequate renal function to be effective and is contraindicated in patients with a creatinine clearance below 50 mL/min or a history of kidney stones [17]. Although probenecid is generally well-tolerated, it increases the risk of kidney stones because of elevated uric acid excretion. Therefore, adequate hydration and urine alkalinization are recommended to prevent stone formation [18].

#### 2.2.4. Lesinurad (Systemic)

Lesinurad is a selective uric acid reabsorption inhibitor that targets the URAT1 transporter in the renal tubules. It is used in combination with a xanthine oxidase inhibitor to enhance urate-lowering efficacy in patients who do not achieve target levels with monotherapy. Although lesinurad has been demonstrated to be effective, it poses a risk of renal adverse events, such as acute kidney injury, particularly when used alone or in patients with preexisting renal impairment [19]. To mitigate this risk, lesinurad should always be used in combination with a xanthine oxidase inhibitor [20].

#### 2.2.5. Tibetan Medicine Qingpeng Ointment (Local)

Qingpeng ointment, a traditional Tibetan herbal remedy, offers a promising alternative for gouty arthritis management. Studies have revealed that Qingpeng ointment significantly reduced pain and inflammation in patients with gout, indicating its efficacy as an adjunct or alternative treatment [24]. The ointment contains herbal components such as *Aconitum carmichaelii* and *Notopterygium incisum*, which inhibit key inflammatory mediators, including TNF-α, IL-1β, and IL-6, thereby reducing acute pain and swelling associated with gout flares [25]. Additionally, Qingpeng ointment promotes blood circulation and the removal of metabolic waste products, providing both symptomatic relief and addressing the underlying inflammation. This ointment has a favorable safety profile and minimal adverse effects, as reported in clinical trials, rendering it a suitable treatment option for long-term management, particularly for patients who prefer natural remedies or those who cannot tolerate conventional therapies [26].

#### 2.2.6. Tongfengkang

Tongfengkang is a traditional Chinese herbal formula that demonstrates the potential for gout management because of its natural composition and multifaceted mechanisms of action. Research indicated that Tongfengkang alleviates inflammation and pain by modulating inflammatory pathways and reducing serum uric acid levels [27]. The formula contains bioactive compounds that inhibit inflammatory cytokines such as TNF-α, IL-1β, and IL-6 and enhance renal excretion of uric acid, providing both symptomatic relief and addressing the underlying causes of gout [28,29]. Clinical trials have reported that Tongfengkang is generally well-tolerated, with a favorable safety profile and minimal adverse effects [30]. Its use may reduce dependence on NSAIDs and corticosteroids, and this potentially minimizes the risk of gastrointestinal, cardiovascular, and renal complications. With further validation, Tongfengkang could become an integral component of the gout treatment regimen, offering a natural and effective alternative for long-term management.

### 2.3. Pharmaceutical Plant Additives

#### 2.3.1. Citrullus colocynthis

*Citrullus colocynthis* (commonly known as bitter apple or bitter cucumber) has garnered considerable research interest as a potential alternative or adjunct therapy for gouty arthritis, due to its anti-inflammatory and uric acid-lowering properties. Studies, such as those by Raziani et al., have demonstrated that a 1-month treatment with *Citrullus colocynthis* significantly reduces pain intensity and serum uric acid levels in patients with gouty arthritis compared to control treatments [34]. These therapeutic effects are attributed to bioactive compounds, including cucurbitacins, flavonoids, and alkaloids, which inhibit key pro-inflammatory mediators like TNF-α, IL-1β, and IL-6—cytokines involved in the inflammatory response during gout attacks [35,36]. By modulating these inflammatory pathways, *Citrullus colocynthis* may help reduce the severity and frequency of gout flares. Additionally, its active compounds promote uric acid excretion and inhibit uric acid production, leading to overall lowered serum uric acid levels [37]. However, despite these promising findings, the use of *Citrullus colocynthis* is accompanied by significant safety concerns. The plant is known for its potent laxative effects even at low doses, which may limit its tolerability, especially in patients with sensitive gastrointestinal systems. Moreover, the narrow margin between therapeutic and toxic doses poses a risk of overdose, which can lead to severe adverse effects or potentially fatal outcomes. Due to these risks, the use of *Citrullus colocynthis* should always be approached with caution and under strict medical supervision [38].

Current studies on *Citrullus colocynthis* are largely limited to small-scale trials with short follow-up periods, making it difficult to fully assess its long-term efficacy and safety. To ensure patient safety, proper dosing guidelines must be established, and patients should be closely monitored for signs of gastrointestinal distress. Given the potential for serious side effects, *Citrullus colocynthis* may not be suitable for all patients, particularly those with pre-existing gastrointestinal conditions or those at higher risk of complications. To better understand the therapeutic potential of *Citrullus colocynthis*, large-scale clinical trials are needed to verify its efficacy and safety across diverse patient populations, determine optimal dosage and formulation, and evaluate its long-term effects [39]. With further research, *Citrullus colocynthis* could potentially become a valuable component of comprehensive gouty arthritis management, offering a complementary option that might reduce the required dosage and frequency of conventional medications, thereby minimizing their associated adverse effects.

#### 2.3.2. Cangzhu

Cangzhu (*Atractylodes lancea*), is known for its anti-inflammatory and analgesic properties; hence, it is a promising alternative or adjunct treatment for gouty arthritis. Li et al. reported that Cangzhu significantly reduced inflammation and pain in patients with gout, suggesting its potential role in managing this condition [61]. The therapeutic effects of Cangzhu are primarily attributed to its bioactive compounds, including atractylenolide I, atractylenolide II, and wogonin, which inhibit key inflammatory pathways and suppress the production of proinflammatory cytokines such as TNF-α, IL-1β, and IL-6 [62]. This mechanism helps alleviate the acute inflammation and pain associated with gout flares [63]. Additionally, Cangzhu enhances antioxidant defenses, which may protect joint tissues from oxidative stress and further inflammation, thus providing both symptomatic relief and potentially mitigating long-term joint damage. The safety and tolerability of Cangzhu render it a promising option for gouty arthritis management [64]. As a natural remedy, Cangzhu is generally well-tolerated with minimal adverse effects, which may reduce the need for high doses of conventional drugs and thus improve patient compliance. Furthermore, Cangzhu has been effectively integrated into existing treatment regimens, either as a standalone therapy or in combination with other treatments, to enhance overall efficacy and patient outcomes [62,63,64]. However, similar to *Citrullus colocynthis*, evidence supporting the use of Cangzhu is primarily derived from preliminary studies. Large-scale RCTs are necessary to establish the long-term efficacy, safety, and optimal use of Cangzhu in diverse patient populations. With advances in research, Cangzhu could become a valuable addition to the holistic management of gouty arthritis.

## 3. Anti-Inflammatory Therapies Targeting the Secondary Inflammatory Cascade

In addition to administering urate-lowering treatments, managing the CARES trial reported that febuxostat use was associated with an increased risk of cardiovascular events, which is crucial in the pathogenesis of gouty arthritis [65]. This inflammation is primarily triggered by the deposition of MSU crystals in the joints, which initiate a robust inflammatory cascade involving the activation of immune cells and the release of proinflammatory cytokines such as IL-1β, TNF-α, and IL-6. Anti-inflammatory therapies aim to alleviate pain and swelling associated with acute gout flares and to prevent recurrent episodes by targeting these underlying inflammatory mechanisms.

### 3.1. Conventional Therapies

Conventional medications, including NSAIDs, corticosteroids, and colchicine, are the primary options for managing acute gout flares by reducing inflammation and pain [7]. NSAIDs work by inhibiting cyclooxygenase enzymes (COX-1 and COX-2), which reduces the production of proinflammatory prostaglandins. Conversely, corticosteroids suppress various inflammatory pathways, including cytokine production, to rapidly reduce inflammation. Colchicine is a plant-based therapy derived from *Colchicum autumnale* (autumn crocus) and has been used for centuries in the treatment of gout. Its effectiveness is attributed to its ability to inhibit microtubule polymerization, which in turn reduces the inflammatory response during gout flares. By targeting the inflammatory cascade, colchicine plays a critical role in reducing pain and swelling in gout patients, particularly during acute attacks. Despite its long history of use, colchicine remains a valuable option for patients, especially those who may not tolerate other anti-inflammatory therapies. However, its use requires careful monitoring due to potential gastrointestinal side effects and the risk of toxicity at higher doses [8]. Despite their efficacy, these therapies are often associated with adverse effects, such as gastrointestinal bleeding, cardiovascular risks, and renal toxicity, particularly with long-term use or in patients with comorbid conditions.

### 3.2. Novel Therapies

In addition to conventional treatments, recent advances have introduced novel and complementary therapies that target the secondary inflammatory cascade in gout by modulating specific immune pathways and inflammatory mediators involved in sustaining and amplifying the inflammatory response following the initial deposition of MSU crystals.

#### 3.2.1. Canakinumab

Canakinumab, an IL-1β inhibitor, represents a promising therapeutic option for patients with gouty arthritis, particularly those who are intolerant or unresponsive to conventional therapies [21]. As a proinflammatory cytokine, IL-1β plays a crucial role in the inflammatory cascade that underlies gout flares. Canakinumab selectively binds to IL-1β, thereby effectively reducing inflammation and preventing acute flares. Clinical studies have demonstrated that canakinumab significantly reduces the frequency and severity of gout flares compared with a placebo, providing rapid and sustained pain relief [22,23]. Additionally, its long half-life allows for less frequent dosing, which enhances patient compliance and convenience. However, the use of canakinumab is not without risks. It has been associated with an increased risk of mild-to-moderate infections, necessitating careful monitoring and management during treatment [66,67]. Moreover, its high cost may limit its accessibility and widespread use in clinical practice. Furthermore, while canakinumab’s efficacy in reducing gout flares is well-established, its use requires particular caution in patients with cytochrome P450 enzyme defects. These patients may have altered drug metabolism and an increased risk of hepatotoxicity, making regular liver function tests essential. Alternative treatments should be considered for those at higher risk of liver-related adverse effects.

Given these factors, additional large-scale, long-term clinical trials are needed to fully understand canakinumab’s sustained benefits, safety profile, and potential applications in diverse patient populations. Despite these challenges, canakinumab holds potential as a valuable component of comprehensive gout management, especially for patients with multiple comorbidities such as cardiovascular disease, hypertension, and chronic kidney disease. By reducing systemic inflammation, canakinumab may offer additional benefits beyond controlling gout flares [68].

#### 3.2.2. Ozone Therapy

Ozone therapy, which involves administering ozone gas to enhance oxygen delivery to cells and reduce oxidative stress, is another potential alternative treatment for gout management [40]. Research conducted using animal models has demonstrated that ozone therapy significantly alleviated symptoms of acute gouty arthritis by inhibiting the NLRP3 inflammasome, a critical component of the inflammatory response in gout [41]. Ozone therapy modulates immune responses by stimulating the production of anti-inflammatory cytokines and reducing the levels of proinflammatory mediators, thus limiting the acute inflammatory response associated with gout flares [42]. Moreover, ozone therapy enhances oxygen metabolism and increases antioxidant production, which can mitigate oxidative stress and protect joint tissues from long-term damage [43]. The safety profile of ozone therapy is generally favorable, with minimal adverse effects reported when administered correctly; therefore, immune response is a viable option for patients seeking alternative or adjunctive treatments [69]. However, more extensive clinical studies are warranted to establish its long-term efficacy, safety, and optimal therapeutic protocols.

#### 3.2.3. Warm Ginger Compress Therapy

Warm ginger compress therapy, which entails applying a warm cloth soaked in ginger extract to the affected area, offers a natural and noninvasive alternative for managing gouty arthritis. Research has demonstrated that this therapy can significantly reduce pain and inflammation; these effects can be attributed to the bioactive compounds in ginger, such as gingerols and shogaols, which possess potent anti-inflammatory and analgesic properties [31]. These compounds inhibit the production of proinflammatory cytokines and enzymes, such as TNF-α, IL-1β, and cyclooxygenase-2, key contributors to the inflammatory response associated with gout [32]. Additionally, the warmth from the compress promotes blood circulation, which aids in the elimination of metabolic waste products and further alleviates inflammation and discomfort. The safety, simplicity, and minimal side effects of warm ginger compress therapy render it an appealing option for long-term management, potentially reducing the reliance on conventional medications [33]. However, further research is necessary to validate its efficacy in larger patient populations and to establish standardized application protocols.

#### 3.2.4. Physical Activity

Incorporating physical activity into treatment regimens for gouty arthritis represents a holistic approach that can improve joint function, reduce inflammation, and enhance overall health outcomes. Regular physical activity, such as brisk walking, swimming, and low-impact aerobics, has been shown to lower serum uric acid levels, reduce the frequency of gout flares, and improve joint mobility and strength [44]. Exercise also stimulates the release of anti-inflammatory cytokines and decreases levels of proinflammatory mediators, thereby alleviating the chronic inflammation associated with gout [45]. Physical activity plays a critical role in weight management, which is essential for preventing and managing gouty arthritis, as obesity is a well-established risk factor for the disease [46]. A systematic review has demonstrated that weight loss achieved through a combination of diet and physical activity significantly reduces serum uric acid levels and the frequency of gout flares, underscoring the importance of lifestyle modifications in gout management [47].

Current guidelines recommend engaging in at least 150 min of moderate-intensity aerobic exercise per week, such as brisk walking, swimming, or cycling, to support overall health and reduce gout symptoms [70]. This activity should be distributed throughout the week in sessions lasting at least 10 min each. In addition to aerobic exercises, muscle-strengthening activities, such as resistance training, should be performed at least twice a week to enhance joint stability and overall physical fitness. Flexibility exercises, including yoga or stretching, are also beneficial for improving joint mobility and preventing stiffness.

However, exercise programs should be tailored to the specific needs and comorbidities of patients to optimize therapeutic outcomes. Activities should be adjusted based on the patient’s fitness level and medical condition to prevent injury and ensure compliance. By integrating appropriately designed exercise regimens into gout management, patients can achieve better control over their symptoms and improve their overall quality of life.

## 4. Lifestyle Modifications for Gout Management

Effective gout management includes not only pharmacological interventions but also lifestyle changes that can reduce the frequency and severity of gout flares [71]. Key modifications such as maintaining a healthy weight, engaging in regular physical activity, and quitting smoking are essential components of a comprehensive gout management plan. These strategies address critical factors contributing to hyperuricemia and inflammation, thereby enhancing the effectiveness of medical treatments and improving overall health outcomes.

### 4.1. Weight Management

Maintaining a healthy weight is crucial for gout management because excess body weight is strongly associated with higher serum urate levels and an increased frequency of gout flares. Weight loss in individuals with overweight or obesity leads to a reduction in serum uric acid levels through a decrease in insulin resistance and an increase in renal uric acid excretion. Studies have reported that a 5–10% reduction in body weight can significantly lower the risk of gout flares and improve overall health outcomes [72]. Gradual, sustained weight loss through a balanced diet and regular exercise is recommended to optimize gout management and prevent obesity-related comorbidities. However, the long-term efficacy of weight management strategies in reducing gout flares remains elusive. Additional studies are warranted to determine the most effective approaches for different patient populations.

### 4.2. Regular Physical Activity

Engaging in regular physical activity provides numerous benefits for gout management, including reducing inflammation, improving joint function, and enhancing cardiovascular health. Moderate-intensity exercise activities, such as walking, swimming, or cycling, are recommended to help prevent gout flares without causing excessive stress on the joints. A study revealed that engaging in at least 150 min of moderate exercise per week can reduce uric acid levels, reduce the risk of comorbid conditions such as hypertension and diabetes, and improve quality of life [70,73]. Additionally, incorporating strength training and flexibility exercises can enhance joint stability and overall physical fitness, thereby supporting long-term gout management. However, although the benefits of physical activity are strongly supported by observational studies, additional RCTs are necessary to determine the optimal type, intensity, and frequency of exercise for patients with gout.

### 4.3. Smoking Cessation

Smoking is associated with increased inflammation and a higher risk of cardiovascular disease, both of which can exacerbate gout symptoms and complicate its management. Patients with gout are strongly recommended to quit smoking in order to alleviate systemic inflammation and the risk of comorbid conditions. Smoking cessation was demonstrated to enhance the effectiveness of other gout treatments and improve overall health outcomes, including a reduction in gout flares and complications [74]. Supportive strategies, such as counseling, nicotine replacement therapy, or medication, may help individuals quit smoking. Although the impact of smoking cessation on gout management is adequately documented, further research is warranted to explore the mechanisms through which smoking influences uric acid metabolism and to assess the long-term benefits of smoking cessation in diverse patient populations.

**Figure 1:**
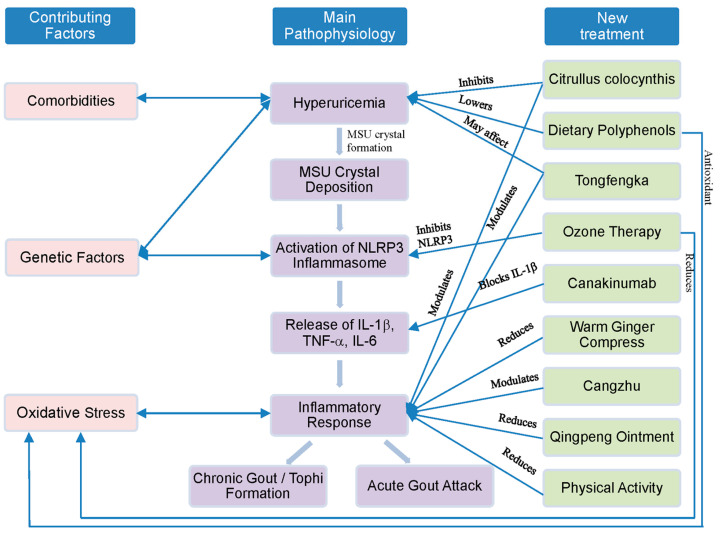
Overview of treatment strategies for gouty arthritis.

A summarized figure illustrating the established and emerging treatment strategies for gouty arthritis has been included to provide a comprehensive visual overview (Figure 1).

## 5. Discussion

The exploration of novel treatment strategies for gouty arthritis in recent years has led to the introduction of several promising approaches aimed at overcoming the limitations of traditional therapies. These innovative treatments, including canakinumab [21,22,23], *Citrullus colocynthis* [34,35,36,37], and ozone therapy [40,41,42,43], exhibit unique mechanisms of action and potentially more favorable safety profiles. Canakinumab, an IL-1β inhibitor, has demonstrated significant efficacy in reducing inflammation and preventing acute gout flares, particularly in patients who are intolerant to conventional medications [21,22,23]. Similarly, natural therapies such as *Citrullus colocynthis* and ozone therapy provide nonpharmacological alternatives that may reduce reliance on conventional drugs, thereby minimizing the risk of adverse effects [34,35,36,37,40,41,42,43]. Incorporating these novel therapies into clinical practice could substantially improve patient outcomes by addressing both urate-lowering and anti-inflammatory needs. In addition to these novel therapies, integrating food polyphenols [52,53,54,55,56,57], traditional Chinese and Tibetan medicines [24,25,26,27,28,29,30], and complementary approaches such as warm ginger compresses [31,32,33] and regular physical activity [44,45,46,47] offers a more holistic approach to gout management. These natural treatments are generally well-tolerated, with minimal side effects, rendering them suitable for long-term use in managing gouty arthritis. For example, polyphenol-rich foods such as cherries and berries have been demonstrated to reduce serum urate levels and reduce the frequency of gout flares [55,56], whereas traditional medicines and therapies help alleviate pain and inflammation, thereby promoting overall health and well-being [24,25,26,27,28,29,30].

Although these emerging treatments show promise, the quality of supporting evidence considerably varies across studies, ranging from rigorously designed randomized controlled trials (RCTs) to preliminary animal studies and observational data, necessitating a critical evaluation of the current literature. For example, although canakinumab has demonstrated efficacy, several relevant studies have limitations such as small sample sizes and short follow-up periods, raising concerns about its long-term safety and efficacy in diverse populations [21,22,23]. Evidence supporting natural therapies such as *Citrullus colocynthis* is primarily derived from small-scale trials with limited control groups, which introduces potential bias [34,35,36,37]. Additionally, the lack of standardization in preparation and dosing across studies complicates comparisons and generalization. Ozone therapy, although promising, is predominantly supported by animal models or small-scale human studies, which limits the ability to draw definitive conclusions about its clinical utility [40,41,42,43]. Studies on traditional Chinese medicine and Tibetan medicine often lack methodological rigor, such as proper blinding and placebo controls, and exhibit heterogeneity in formulations and treatment protocols, further complicating the interpretation of results [24,25,26,27,28,29,30]. Data on lifestyle modifications and dietary interventions are encouraging but are largely observational and subject to confounding factors, which limits the ability to make causal inferences [44,45,46,47].

Studies on emerging gout therapies have several methodological limitations. Numerous trials, particularly those investigating newer treatments, have insufficient sample sizes, impairing the ability to detect clinically meaningful differences or rare adverse events [21]. Short follow-up periods further constrain our understanding of long-term efficacy and safety profiles, which is essential for managing a chronic condition such as gout. Limited diversity in study populations may affect the generalizability of findings to broader patient groups, including those with common comorbidities associated with gout. Additionally, variability in outcome measures across studies complicates direct comparisons, underscoring the need for standardization of core outcome sets in gout research [75,76]. The potential for publication bias is another concern, as negative results are less likely to be published, which may distort the overall perception of treatment efficacy. To address these limitations and strengthen the evidence base for emerging gout therapies, future research should prioritize large-scale, long-term RCTs with diverse patient populations to validate safety and effectiveness. Standardizing treatment protocols, particularly for natural and traditional therapies, is crucial for improving reproducibility and comparability of results [34,35,36,37]. Implementing core outcome sets in gout research will facilitate meta-analyses and evidence synthesis. Furthermore, head-to-head comparisons between new and established therapies are necessary to determine relative efficacy and safety. Mechanistic studies aimed at elucidating the molecular pathways underlying the effects of promising therapies, particularly for natural and complementary treatments, will provide valuable insights for optimizing their use [22,23].

Patient compliance is a crucial factor influencing the effectiveness of gout treatment regimens [75,76,77]. The complexity and side effects of traditional treatments often result in poor adherence, thereby diminishing their therapeutic outcomes [78,79]. Newer strategies, such as warm ginger compress therapy and increased physical activity, provide simpler and more manageable alternatives that patients can easily integrate into their daily routines. These patient-centered strategies not only improve compliance but also reduce flare frequency and improve quality of life. When used in combination with existing ULTs, these treatments provide a comprehensive management strategy that balances efficacy with minimal side effects. While emerging treatments for gouty arthritis show significant promise, large-scale, long-term randomized controlled trials (RCTs) are needed to confirm their efficacy and safety across diverse patient populations. Comparative studies between new and traditional therapies, as well as investigations into combination treatments, could offer valuable insights for optimizing gout management. Understanding the molecular mechanisms underlying these therapies may also reveal potential synergies with conventional treatments, paving the way for personalized medicine approaches.

Genetic factors play a crucial role in individual responses to gout treatments, influencing both their efficacy and safety. Variations in genes such as SLC2A9 and ABCG2, which encode key urate transporters, can significantly affect uric acid metabolism and excretion, thereby impacting the effectiveness of urate-lowering therapies [10]. Additionally, the SLC22A12 gene, which encodes the URAT1 transporter, is vital for renal urate reabsorption. Variants in SLC22A12 have been linked to altered urate handling, contributing to hyperuricemia and increasing the risk of gout. Mutations or polymorphisms in this gene can significantly influence the efficacy of urate-lowering therapies, particularly uricosuric agents that target the URAT1 transporter. Incorporating genetic screening for SLC22A12 variants into clinical practice may enable more personalized treatment strategies, optimizing the selection of uricosuric drugs and improving outcomes in gout management. Genetic screening for these variants may help tailor treatment plans to achieve optimal uric acid levels. Similarly, the presence of the HLA-B58:01* allele is a known risk factor for severe hypersensitivity reactions to allopurinol, making pre-therapy genetic testing valuable [13]. Additionally, variations in cytochrome P450 enzymes, such as CYP2C9 and CYP3A4, can influence the metabolism of medications like febuxostat and canakinumab, potentially altering their efficacy and risk profiles [80]. By incorporating genetic information into clinical practice, clinicians can personalize treatment strategies, improve therapeutic outcomes, and minimize adverse effects.

Looking ahead, future research should focus on integrating these novel therapies into existing treatment guidelines, with an emphasis on personalized medicine. Developing protocols for combining these treatments with standard care, optimizing drug delivery systems, and leveraging technological advancements, such as AI-driven treatment algorithms, will be crucial for enhancing therapeutic outcomes. Interdisciplinary collaboration among rheumatology, pharmacology, and traditional medicine will be essential to advance comprehensive research efforts. Additionally, economic evaluations will be necessary to assess the feasibility and cost-effectiveness of these therapies in various healthcare settings. Collectively, these initiatives will help shift the field toward more effective, personalized management strategies for gouty arthritis, revolutionizing treatment paradigms to enhance patient outcomes and reduce adverse effects.

## 6. Conclusions

In conclusion, managing gouty arthritis requires a multifaceted approach that integrates both traditional and emerging therapeutic strategies. Conventional therapies that include NSAIDs, corticosteroids, colchicine, and urate-lowering medications such as allopurinol and febuxostat are still essential for managing acute flares and reducing serum urate levels. However, their limitations highlight the need for new approaches. Recent advances in the understanding of the biochemical pathways and molecular mechanisms underlying gout have led to the development of innovative therapies targeting uric acid metabolism, inflammation, and crystal formation. These innovations include pharmaceutical plant additives (e.g., *Citrullus colocynthis* and *Atractylodes lancea*), novel anti-inflammatory agents (e.g., canakinumab and ozone therapy), and complementary treatments (e.g., warm ginger compresses and Qingpeng ointment). Furthermore, lifestyle modifications such as weight management, regular physical activity, smoking cessation, adequate hydration, and dietary changes are vital components of a comprehensive gout-management strategy. These nonpharmacological interventions can enhance the effectiveness of medical treatments and improve overall patient outcomes. Integrating these new therapeutic options, supported by further research and clinical trials, could provide more personalized and effective management for patients with gouty arthritis. Future studies should focus on validating these approaches and assessing their long-term impact on disease progression, quality of life, and comorbidity management, with the ultimate aim of reducing the burden of gout and enhancing patient care.

## Figures and Tables

**Table 1:** Summary of treatment methods for gouty arthritis.

Treatment Strategy	Type	Mechanism of Action	Study Type	Dose/Duration	Percentage Reduction in Uric Acid Levels	Toxic Doses/Side Effects	Advantages	Disadvantages	References
Urate-Lowering Therapies	Allopurinol	Inhibits xanthine oxidase, reducing uric acid production	Clinical Trial	100–800 mg/day, long-term use	35–50%	Risk of hypersensitivity reactions, especially in patients with HLA-B*5801	Effective in reducing serum urate levels; well-studied	Requires dose adjustments for renal impairment; potential for severe side effects	[13,14]
	Febuxostat	Selective inhibition of xanthine oxidase, lowers uric acid levels	Clinical Trial	40–120 mg/day, long-term use	40–60%	Potential increased cardiovascular risk	More effective in patients intolerant to allopurinol	Costlier than allopurinol; cardiovascular concerns	[15,16]
	Probenecid	Increases renal excretion of uric acid	Clinical Trial	500–2000 mg/day, long-term use	20–30%	Risk of kidney stones; contraindicated in patients with renal impairment	Increases uric acid excretion	Requires good renal function; risk of kidney stones	[17,18]
	Lesinurad	Blocks URAT1 transporter to enhance urate excretion	Clinical Trial	200 mg/day, in combination with xanthine oxidase inhibitors	25–35% (when combined with xanthine oxidase inhibitors)	Risk of renal adverse events, such as acute kidney injury	Effective in combination with xanthine oxidase inhibitors	Should not be used as monotherapy; requires combination with other agents	[19,20]
Anti-Inflammatory Therapies	Canakinumab	Inhibits IL-1β to reduce inflammation and prevent flares	Randomized Controlled Trial	150 mg subcutaneous injection, every 8 weeks	Not applicable	Mild to moderate infections; high cost	Effective for patients intolerant to conventional treatments	High cost may limit use; requires further studies for long-term safety	[21,22,23]
Complementary Therapies	Qingpeng Ointment	Reduces inflammation by inhibiting TNF-α, IL-1β, IL-6	Clinical Trial	Topical application, 2–4 weeks	Not applicable	Rare skin irritation, allergic reactions	Natural and well-tolerated	Limited data from large-scale studies	[24,25,26]
	Tongfengkang (Herbal Formula)	Modulates inflammatory pathways and enhances urate excretion	In vitro, in vivo, Clinical Trial	Varies by formulation	Approx. 20%	Generally well-tolerated; mild gastrointestinal discomfort at high doses	Reduces inflammation and urate levels	Evidence primarily from small-scale studies; variability in formulations	[27,28,29,30]
	Warm Ginger Compress Therapy	Inhibits proinflammatory cytokines (TNF-α, IL-1β, COX-2)	Observational Studies	Applied 2–3 times daily, 7–14 days	Not applicable	Minimal side effects; rare skin irritation	Easy to use; safe for long-term use	Limited to observational studies; requires more clinical validation	[31,32,33]
Phytotherapy	*Citrullus colocynthis*	Inhibits proinflammatory cytokines and promotes uric acid excretion	In vitro, in vivo Studies	300 mg/kg/day, 14–28 days	15–25%	Gastrointestinal upset at high doses	Natural alternative with potential fewer side effects	Requires standardization in preparation and dosing; limited to small-scale trials	[34,35,36,37,38,39]
Alternative Therapies	Ozone Therapy	Modulates immune response and reduces oxidative stress	Animal Models, Small-scale Human Studies	30–50 μg/mL, 3 times/week	Not applicable	Potential for oxidative damage at high doses	Promising in reducing acute gout symptoms	Limited clinical evidence; mostly supported by animal studies	[40,41,42,43]
Lifestyle Modifications	Physical Activity	Reduces serum uric acid levels; stimulates anti-inflammatory pathways	Observational Studies	At least 150 min of moderate exercise per week	Approx. 10–20%	Generally safe; may not be suitable for all patients with joint issues	Improves overall health; decreases flare frequency	Requires tailored exercise programs; compliance may vary	[44,45,46,47]

HLA: human leukocyte antigen; URAT1: urate transporter 1; IL-1β: Interleukin-1 beta; TNF-α: tumor necrosis factor-alpha, IL-6: interleukin-6, COX-2: cyclooxygenase 2.

## Data Availability

Not applicable.
